# Antimicrobial Peptides Demonstrate Activity against Resistant Bacterial Pathogens

**DOI:** 10.3390/idr15040046

**Published:** 2023-08-14

**Authors:** Mary Garvey

**Affiliations:** 1 Department of Life Science, Atlantic Technological University, F91YW50 Sligo, Ireland; mary.garvey@atu.ie; 2 Centre for Precision Engineering, Materials and Manufacturing Research (PEM), Atlantic Technological University, F91YW50 Sligo, Ireland

**Keywords:** antimicrobial peptide, potent, therapeutic, resistance, infectious disease

## Abstract

The antimicrobial resistance crisis is an ongoing major threat to public health safety. Low- and middle-income countries are particularly susceptible to higher fatality rates and the economic impact of antimicrobial resistance (AMR). As an increasing number of pathogens emerge with multi- and pan-drug resistance to last-resort antibiotics, there is an urgent need to provide alternative antibacterial options to mitigate disease transmission, morbidity, and mortality. As identified by the World Health Organization (WHO), critically important pathogens such as *Klebsiella* and *Pseudomonas* species are becoming resistant to last-resort antibiotics including colistin while being frequently isolated from clinical cases of infection. Antimicrobial peptides are potent amino acid sequences produced by many life forms from prokaryotic, fungal, plant, to animal species. These peptides have many advantages, including their multi-hit mode of action, potency, and rapid onset of action with low levels of resistance being evident. These innate defense mechanisms also have an immune-stimulating action among other activities in vivo, thus making them ideal therapeutic options. Large-scale production and formulation issues (pharmacokinetics, pharmacodynamics), high cost, and protease instability hinder their mass production and limit their clinical application. This review outlines the potential of these peptides to act as therapeutic agents in the treatment of multidrug-resistant infections considering the mode of action, resistance, and formulation aspects. Clinically relevant Gram-positive and Gram-negative pathogens are highlighted according to the WHO priority pathogen list.

## 1. Introduction

**Figure 1:**
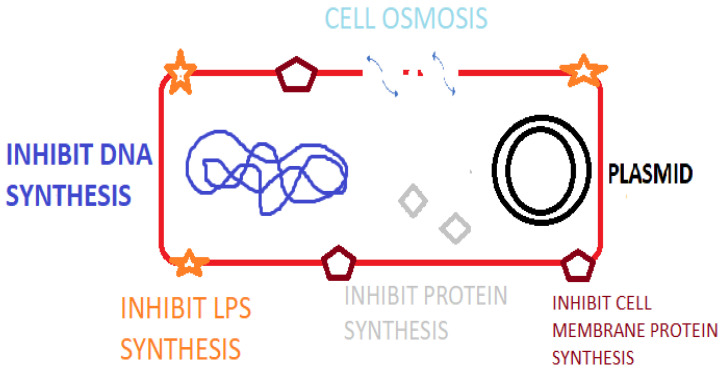
Mode of action of AMPS against bacterial pathogens.

Antimicrobial peptides (AMPs) are peptide sequences produced by bacteria, archaea, protozoal, fungal, amphibians, birds, fish, plant, and animal species. In animal and plant species, AMPs are a component of innate immunity to infectious agents [1] as listed in the Antimicrobial Peptide Database (APD). Additionally, there are numerous synthetically synthesized AMPs [2]. AMPs possess potent broad-spectrum activity against bacteria, fungi and viral species [3] and play important roles in immune regulation via agonizing cell receptors [4], healing and possess antitumor activity [5]. Certain animal AMPs have a chemotactic action on leukocytes, regulate cell proliferation, epithelialization, angiogenesis, wound healing, and adaptive immunity [2]. As a component of the innate immune system, AMPs offer a more rapid rate of production and antimicrobial action than the acquired immune response utilizing immunoglobulins [5]. AMPs contain between 10 and 60 amino acids and typically average ca. 33 amino acids in length [6]. Specific genes code for AMPs, which are induced by external factors leading to gene expression and amino acid production [3]. The mode of action of AMPs relates to osmotic lysis due to interaction with the bacterial membrane. Research also demonstrates that AMPs result in membrane damage, inhibit macromolecular synthesis, damage cellular organelle and DNA, inhibit enzymes and regulate host immunity [7] (Figure 1). Inhibiting essential cellular process such as DNA replication, nucleic acid synthesis, protein synthesis, liposaccharide (LPS) and cell wall formation is possible with AMPs that cross the cytoplasmic membrane [8]. The presence of a cationic rich moiety and hydrophobic aminos acids is typical in AMPs, leading to a cationic arrangement with amphiphilic (hydrophilic and hydrophobic) characteristics [3]. The overall positive charge of AMPs promotes binding to the bacterial cell membrane via electrostatic interactions with the negatively charged phospholipid components (phosphatidylglycerol, cardiolipin, or phosphatidylserine) [9]. This allows for specificity to prokaryotic membranes because mammalian cells have a net-neutral charge due to the presence of zwitterionic phospholipids, e.g., phosphatidylethanolamine, phosphatidylcholine, or sphingomyelin [10]. Nisin, for example, is a ribosomal-synthesized bacteriocin produced by *Lactococcus lactis* having antibacterial activity against Gram-positive species including *Staphylococci*, *Streptococci*, *Listeria*, *Bacilli* and *Enterococci* species [11]. Indolicidin, for example, is a bovine cathelicidin AMP that has activity by disrupting the bacterial cell membrane and by inhibition of DNA topoisomerase synthesis [12]. AMPs can be classified based on their activity, structural characteristics, amino acid-rich species, and source host [6].

Currently, AMPs are classified into five major families based on their structural compositions and amino sequence: defensins, cathelicidins, hepcidins, histone-rich peptides (Table 1), and the fish-specific piscidins [13]. Mammals possess two classes of AMPs, the cathelicidins and defensins, with a third family termed the histatins also found in humans [1]. Defensins are furthered categorized as α-, β-, and θ-defensins depending on the position of disulphide bonds [9]. Human β-defensins are expressed by epithelial cells, monocytes, macrophages, and dendritic cells where they actively regulate the microbiome and dysbiosis [14]. The fish-specific AMP piscidin1 has broad-spectrum antimicrobial activity and regulate inflammatory and immune responses [15]. In animals, the AMPs are stored in the granules of granulocytes, e.g., neutrophils and macrophages [16]. Defensins and cathelicidins are also present in animal milk (breast milk in humans) with the concentrations of each varying in colostrum versus mature milk [17]. As such, the provision of breast milk to newborns offers protection against neonatal infection, e.g., necrotizing enterocolitis, infection of the gastrointestinal tract (GIT), and respiratory infections [17]. Plants possess numerous AMPs isolated from the stems, seeds, and leaf subsequently categorized into thionins, defensins, and snakins [6]. Insect AMPs (e.g., cecropin) are produced in cells and fat bodies and have demonstrated antimicrobial, anti-inflammatory activity and in some cases anticancer activity [18]. Hepcidin, a cysteine-rich AMP, has an important role in iron regulation and antimicrobial, anticancer, antiparasitic, and immunomodulatory activity [19]. As a peptide hormone, hepcidin is produced and secreted by liver hepatocytes and Kupffer’s cells [20]. Human hepcidin displays moderate antimicrobial activity [4]. Bacteriocins are antimicrobial peptides produced by bacterial species, which are cationic and smaller than 10 kDa (excluding class III bacteriocins) [11]. The small size and cationic nature of bacteriocins allows for adherence and penetration of bacterial phospholipid membranes resulting in cell death [21]. Bacteriocins exhibit multiple modes of action by forming pores in membranes, inhibiting cell wall biosynthesis, and affecting cellular respiration [11].

Currently, there is a pandemic of antimicrobial-resistant (AMR) and extensively drug-resistant (XDR) bacterial pathogens, where AMR species are predicted to cause increased mortality rates yearly. The widespread application of antibiotics in clinical settings, animal husbandry, and food production (agriculture and aquaculture) has proliferated the emergence and re-emergence of AMR pathogens. Species including vancomycin-resistant *Enterococcus* (VRE) and methicillin-resistant *Staphylococcus aureus* (MRSA) and the ESKAPE pathogens (*Enterococcus faecium*, *Staphylococcus aureus*, *Klebsiella pneumoniae*, *Acinetobacter baumannii*, *Pseudomonas aeruginosa*, and *Enterobacter* species) as well as AMR fungal species are commonly identified in difficult-to-treat cases of infectious disease [22]. Small colony variants of *S. aureus* are associated with persistent difficult-to-treat skin infections [14], while *S. aureus* bacteremia is estimated to be fatal in approximately 30% of patients. The application of AMPs in the treatment of infectious disease and as biocontrol agents in food production may offer solutions to mitigating the threat of AMR pathogens. Replacing antibiotics and antimicrobial pesticides has become important in the pharmaceutical, agricultural, veterinary, and food industries given the focus on green technologies aligned with the Sustainability Development Goals (SDGs) [23]. Importantly, antibacterial peptides currently applied therapeutically include gramicidin (for topical and eye application) and colistin (polymyxin E) [24]. This review outlines the application of AMPs against clinical pathogens associated with high morbidity with a focus on mitigating AMR pathogens.

**Table 1:** Examples of antimicrobial peptides from varying sources in terms of their antibacterial action and relevant additional information.

Family	AMP	Action	Additional Info
Defensin	Alpha- and beta-defensins Human neutrophil peptides (HNPs) [25] Human enteric defensins	Directly kill phagocytosed microbes. Can enhance the production of inflammatory cytokines, e.g., interleukin-1 [25]	Produced by neutrophils, lymphocytes, and epithelial cells of the skin and mucous membranes [1]. Stored in the azurophilic granules of human neutrophils. Secreted by paneth cells in the small intestinal
Cathelicidins	Bactenecins Human cathelicidin LL-37 [9]	Immunomodulatory activity, impacts quorum sensing mechanisms in *P. aeruginosa* biofilm formation [25], apoptosis induction, inflammasome activation, and phagocytosis [13] LL-37 inhibits *Aspergillus fumigatus* infection and reduces inflammation [9], LL37 analogue peptides (AC-1, AC-2, LL37-1, and D) effetive against *C. albicans* yeasts [3]	The bactenecin ChBac3.4 appears more active against bacterial membranes and tumor cells compared to normal cells [26] LL-37 effective against various Gram-positive and Gram-negative bacteria, and AMR species [9]
Hepcidins	Human liver-expressed antimicrobial peptide (LEAP) Fish hepcidins HAMP1 and HAMP2	Antibacterial Iron regulatory mode of action [19]	Type II acute phase protein Results in a reduction in ferroportin expression
Histone- histidine-rich peptides	Histatins—cationic peptides secreted into human saliva by salivary glands	Antifungal action, antibacterial [27]	High biocompatibility, effective against azole-resistant fungi [27]
Piscidins	Present mainly in the tissues of gills, muscle, head kidney, skin, and intestine [15]	Potent and broad-spectrum [15], antibacterial, antifungal, and antiviral properties	Efficacy toward MDR MRSA, vancomycin-resistant *Enterococci*, piscidin has antitumor activity against cancer-derived cell lines [15]

HNPs—human neutrophil peptides, AMR—antimicrobial resistance, MDR—multidrug resistant, LEAP—human liver-expressed AMP, MRSA—methicillin-resistant S. aureus.

## 2. Clinical Bacterial Infectious Disease

The application of antimicrobial agents and vaccination programs have provided excellent disease prevention and control strategies for decades. The emergence and proliferation of antimicrobial resistance, however, have greatly hindered this approach, where pan-drug-resistant bacteria are often isolated from cases of infectious disease [28]. Morbidity rates resultant from infectious disease are increasing globally, particularly in developing countries. Factors including demographic location, emergence, and re-emergence of pathogenic microbes, AMR, population growth, climate change, and globalization are contributing to disease transmission. Importantly, AMR promotes biocidal resistance with many clinical pathogens displaying intrinsic and acquired resistance to many antimicrobial chemicals and antibiotic therapeutics. Extended-spectrum beta-lactamase (ESBLs) producing Enterobacterales including *K. pneumoniae* and *Escherichia coli* are now frequently isolated from clinical infections in tertiary care facilities including intensive care units (ICUs) [29]. Importantly, pneumonia caused by MDR Gram-negative bacteria is associated with morbidity and high mortality rates, particularly in ICU patients [30]. Enterobacterales are Gram-negative facultative anaerobes associated with severe clinical infections including septicemia, community-acquired infections, urinary tract infections (UTIs), and intra-abdominal infections [28]. Gram-negative bacteria including *Acinetobacter* sp., *P. aeruginosa*, *E. coli*, and *Klebsiella* sp. are frequent causative agents of UTIs, with *S. aureus*, coagulase negative staphylococci, and *Enterococcus* species being common Gram-positive agents of infection with resistance species resulting in high mortality rates annually [31]. Bloodstream infections (BSIs) resultant from infections of the lungs, abdominal cavity, and genitourinary track are associated with high mortality in North America and Europe [32]. Approximately 60% of bacteria detected in BSIs display resistance to third-generation cephalosporins, including ceftriaxone and ceftazidime with ≥50% of *E. coli* isolates displaying resistance to fluoroquinolones [33]. Additionally, carbapenem resistance is also emerging in Enterobacterales species, with an increasing prevalence in *K. pneumoniae* and *E. coli* where there is increased risk of morbidity [34]. *K. pneumoniae* is responsible for approximately 30% of Gram-negative nosocomial and community-acquired infections globally [35]. Bacterial pathogens were the second-leading cause of mortality in 2019 and were associated with one in eight deaths globally. A study published in 2022 identified five pathogens that were associated with 750,000 or 50% of these bacterial deaths, namely *S. aureus*, *E. coli*, *Streptococcus pneumoniae*, *K. pneumoniae*, and *P. aeruginosa* [36]. *S. aureus* was the most frequent causative agent of deaths, totaling 1.1 million mortalities with *E. coli* resulting in 950,000 deaths, *S. pneumoniae* in 829,000, *K. pneumoniae* in 790,000, and *P. aeruginosa* resulting in 559,000 deaths [36]. The antibiotic colistin has been the drug of choice for Gram-negative bacterial infections of species such as *P. aeruginosa*, *A. baumannii*, *Klebsiella* sp., *E. coli*, and other Enterobacterales due to its action on the liposaccharide (LPS) of the outer membrane [35]. MDR species of *K. pneumoniae* displaying ESBL, carbapenem, cephalosporin, fluoroquinolone, and aminoglycoside resistance are currently treated with the antibiotics tigecycline and colistin [31]. Colistin-resistant *K. pneumoniae* have now emerged due to the overuse and misuse of colistin both in clinical and veterinary settings [28]. Furthermore, colistin has biocompatibility issues due to its nephrotoxic and neurotoxic adverse side effects due to permeabilization and lysis of eukaryotic membranes [35]. Biocompatibility can be improved via administration as the prodrug colistin methanesulfonate, which produces colistin after enzymatic hydrolysis [37]. The selectivity of AMPs to bacterial pathogens over animal cells relates to the cell membrane with Gram-positive bacteria having varying levels of peptidoglycan, phosphatidylglycerol, and phosphatidylethanolamine, and Gram-negative bacteria having lipoteichoic acids and lipopolysaccharides on the cell surface [38]. The presence of biocompatibility issues raises concerns in for AMP formulation.

There is an urgent need for alternative therapeutic options in the treatment of bacterial infectious disease as stand-alone or combination modalities as well as optimal disinfection agents and protocols. Combination therapy may also allow for the treatment of numerous infectious agents in one treatment protocol [28]. The mitigation of infectious disease and the control of infectious agents fall under SDG 3, “ensure healthy lives and promote wellbeing for all ages”, and are of paramount importance to curb epidemic and pandemic disease outbreaks. The application of AMPs as a method of disease prevention has shown efficacy in the treatment of bacterial pathogens associated with clinical disease as demonstrated by colistin.

### 2.1. AMP Activity against Gram-Positive Pathogens Assoicated with Infectious Disease

Bacterial-produced bacteriocins such as lantibiotics, e.g., nisin, have demonstrated efficacy against many Gram-positive pathogens, including MDR species of MRSA, VRE, vancomycin intermediate *S. aureus* (VISA), *Streptococcus pneumoniae*, *Clostridium*, *Listeria,* and *Bacillus* sp. [11]. Following AMPs’ interaction with the bacterial surface, they must translocate to the cytoplasm and cytosol where they can interrupt cellular activity and metabolic functions resulting in bacteriostatic or bactericidal effects [39]. Nisin has a low minimum inhibitory concentration (MIC) in the nanomolar range, making it have a quite potent bactericidal effect by inducing pores in the bacterial membrane and inhibiting cell wall synthesis via binding to lipid II [40]. Importantly, bacteriocins produced by *Lactobacillus fermentum* present in breast milk a displayed antibacterial action against UTI strains of *Staphylococcus aureus*, MRSA, and *Enterococcus* sp. in vitro [41]. The bacteriocin mutacin-1140 (22 amino acids) produced by *Streptococcus mutans* inhibits peptidoglycan synthesis by inhibiting DNA replication and protein synthesis in clinically important VRE, and *S. aureus* with lysostaphin, a bacteriocin produced by *Staphylococcus simulans* having low human toxicity, has efficacy against *S. aureus* via targeting the pentaglycine bridges in the cell wall [2]. Bacitracin produced by *Bacillus licheniformis* demonstrated antibacterial action against Gram-positive bacteria with an MIC of 2–4 µM [42]. Bacitracin is applied as a topical agent alone or in combination with neomycin and polymyxin B. Furthermore, the ribosomal-produced lantibiotic bacteriocins Pep 5 and epidermin produced by *Staphylococcus epidermidis* display anti-adhesin action against *Staphylococcus* species on surfaces relevant to medical devices that are commonly associated with biofilm colonization [11]. Bacterial biofilm communities display increased resistance to antimicrobials, biocides, and host immune defenses due to decreased bacterial metabolic activity and genetic alterations [43], posing a significant risk particularly with implanted medical devices. The use of the synthetic AMP E6, a 12-amino-acid peptide to coat catheters, prevented infectious disease in mouse models [9]. The AMP melimine has been covalently bound to contact lens surfaces using 1-ethyl-3-[3-dimethylaminopropyl] carbodiimide hydrochloride, reducing the prevalence of lens-associated eye infection [44].

Bacteriocins nisin and the broad-spectrum enterocin (produced by *E. faecalis*) have sporicidal activity against spores of *Clostridium botulism*, *B. cereus,* and *Geobacillus stearothermophilus* [11]. The efficacy of bacteriocins can be further improved via a combination approach. Lacticin 3147, for example, consists of lantibiotics Ltnα and Ltnβ and possess efficacy against MRSA, VRE, *Pneumococcus*, *Streptococcus*, *Clostridium,* and *Mycobacterium* species [45]. Thusin is a two-component bacteriocin produced by *Bacillus thuringiensis* containing Thsα and Thsβ, and it demonstrated efficacy against *B. cereus*, *B. thuringiensis*, *B. subtilis*, *B. pumilus*, *E. faecalis*, *L. monocytogenes*, *S. aureus*, and *S. pneumoniae* [45]. In vitro studies using the bacteriocin AS-48 produced by *Enterococcus faecalis* demonstrated efficacy against *Mycobacterium tuberculosis*, with pentocin being effective against MDR *S. aureus* and entianin and enterocins DD28 and DD93 being effective against MRSA and VRE reference strains [46]. The combination application of nisin with antibiotics penicillin, ciprofloxacin, and chloramphenicol has demonstrated antibiofilm activity against *E. faecalis* [47]. Nisin Z in combination with novobiocin demonstrated efficacy against *S. aureus*, *S. epidermidis,* and the Gram-negative *E. coli* [48]. This synergistic effect or augmentation allows for lower doses of antibiotics while producing the therapeutic effect [49].

The AMP colistin produced by *Paenibacillus polymyxa* subspecies *colistinus* has nephrotoxic side effects when applied as an antibacterial therapeutic [24]. Colistin resistance is now evident in many pathogens, namely in *E. coli* due to the presence of MCR genes, which may be spread via horizontal gene transfer (HGT) among species [28,31]. The cyclic lipopeptide daptomycin (having 13 amino acids) having an ester in its structure is produced by *Streptomyces roseosporus* and is currently listed as a last-resort antibacterial for the treatment of Gram-positive infections (Table 2) having activity via membrane permeabilization and leakage [6]. Daptomycin has immunomodulating effects due to its lipopeptide structure and ability to enter neutrophils and macrophages and may impact cytokine activity in the patient [50]. Resistance to daptomycin has emerged resultant from altered membrane composition by *S. aureus* and *B. subtillis* [6]. AMPs’ nikkomycin isolated from *Streptomyces tendae* and *Streptomyces ansochromogenes* and polyoxins isolated from *Streptomyces cacaoi* also demonstrated antifungal activity against *C. albicans* [2]. Nikkomycin is a competitive inhibitor of chitin synthases having activity against fungal *Histoplasma capsulatum*, *Candida* species, and *A. fumigatus*. Pexiganan, which is isolated from frog skin, is the first animal AMP having Gram-positive and Gram-negative bactericidal action [51] to reach phase III clinical studies (ClinicalTrials.gov Identifier: NCT01590758), but it failed to pass phase III trials [42]. The AMPs AG-30, AG-30/5C, WRL3, melimine, 73c, and D-73 demonstrated antibiofilm activity [42]. The human-produced eosinophilic cationic AMP displays activity against Gram-positive species. In vivo animal studies demonstrated that the human neutrophil peptide (HNP)-1 in combination with isoniazid and rifampicin was toxic to *M. tuberculosis* at lower MICs, reducing bacterial infection in the lungs, liver, and spleen [49]. The human cathelicidins LL-37 (having 37 amino acids) is under phase II trials as a topical treatment that has antibacterial and anti-inflammatory action on diabetic foot ulcers (ClinicalTrials.gov Identifier: NCT04098562). Meanwhile, bovine omiganan, which is a cathelicidin AMP, reached phase III clinical trials for potent activity against Gram-positive polysaccharide cell content [38]. Omiganan is theorized to translocate the bacterial membrane and target intracellular molecules such as DNA [12], but omiganan also failed to pass phase III clinical trials [42] (ClinicalTrials.gov Identifier: NCT00231153).

Defensins produced by insects (e.g., defensin like peptide 4) appear to have selective activity against Gram-positive species over Gram-negative [32]. Insect AMPs, however, have limited antibacterial activity and are cytotoxic to human cells, which impedes their clinical application [51,52]. Interestingly, the bacteriocins bactofencin and laterosporulin have similar structure to defensins, with the presence of a disulfide bond and highly conserved cysteine residues imparting a cationic nature [46]. The histatin AMPs, which have high histidine amino acid content, were originally isolated from human salivary glands (50–425 µg/mL) and have demonstrated antibacterial and antifungal activity against *Candida albicans* [27]. Human histatins are AMPs rich in histidine, while AMPs that are not rich in histidine are found in amphibians [4]. AMPs with high histidine peptide content possess good stability and high cell selectivity, which encourages their in vivo application [53]. Porcine protegrin-1 (PG-1) has demonstrated potent antimicrobial activity and cytotoxicity, which has a hemolytic action that limits its therapeutic application, modifying the histidine content, although it may improve its biocompatibility [54]. Research studies report that leucine-rich AMPs, e.g., temporins, appear more toxic to Gram-positive pathogens, with 138 leucine-rich AMPs and 15 proline-rich AMPs, e.g., arasin 1 active against *S. aureus* [4]. Temporins are hydrophobic, C-terminally a-amidated peptides produced by frog species [55]. Temporin A inhibits the release of inflammatory molecules such as TNF-alpha, IL-6, and nitric oxide (NO) by macrophages in mouse models and is antibacterial to AMR Gram-positive species [55]. High amounts of proline-rich AMPs found in mammals are cathelicidins peptides, e.g., Bac5, Bac7, and PR-39 [56]. Studies on mice models have demonstrated that an absence of cathelicidins and defensin AMPs increases susceptibility to *Streptococcal* and *Staphylococcal* infections [52]. The synthetic peptide AMC-109 (LTX-109) inspired by lactoferricin has shown efficacy against dermal infections, impetigo, and nasal infections and antibiofilm activity against Gram-positive *Staphylococcus* species [43]. The bovine cathelicidin AMP BMAP-28 demonstrated efficacy against *E. faecalis* and *S. aureus* independently and in conjunction with vancomycin in animal models of ureteral stent infection and inhibited TNF-alpha release and NO production [55].

**Table 2:** Last-resort antibiotics and species where resistance has been identified.

Last-Resort Antibiotic	Mode of Action/Mode of Resistance	Resistant Species	Infectious Disease
Carbapenems, e.g., meropenem	beta-lactams/metallo-beta-lactamases	*K. pneumoniae*	UTIs, BSIs
Polymyxins, e.g., polymyxin B, colistin	Disruptions of LPS/efflux pump, capsule formation, alteration of LPS [57]	*K. pneumoniae*, *E. coli*, *P. aeruginosa*, *Salmonella typhimurium* [57]	
Aztreonam	Inhibition of cell wall synthesis/chromosomally encoded mutations	Inactive against Gram-positive bacteria, resistance seen in *P. aeruginosa* [58]	Cystic fibrous *P. aeruginosa* lung infection [58]
Cephalosporins of 4th, 5th generation	Beta-lactam rings bind to the penicillin-binding protein and inhibit cell wall formation [59]/hindered by production of beta lactamases	4th gen, e.g., cefepime, has activity against beta-lactamase expressing bacteria, 5th gen, e.g., ceftaroline active against methicillin-resistant *Staphylococci* and penicillin-resistant pneumococci [59]	Cefepime has high activity against Enterobacteriaceae that resistant to third-generation cephalosporins, *Salmonella* ESBL-producing infections [60]. Ceftaroline used to treat osteomyelitis and acute bacterial skin and skin structure infections [61]
Tigecycline	3rd generation tetracycline. glycylcycline antibiotic [62], inhibits protein translation [63]/hindered by production of beta lactamases	Potent action against Gram-positive and Gram-negative bacteria except *Proteus* and *Pseudomonas* [62], resistance observed in *Klebsiella* [63]	Skin and skin structure infections, complicated intra-abdominal infections, and community-acquired pneumonia (CAP) [63]
Fosfomycin	Irreversibly inhibits the initial phase of microbial cell wall synthesis [64]/MurA mutations	Active against Gram-positive and Gram-negative bacteria, e.g., vancomycin-resistant enterococci, MRSA, and carbapenem-resistant Enterobacteriaceae. Resistance seen in *Acinetobacter* species [64]	Treatment or severe soft tissue infections (STIs) in ICUs, serious systemic infections, e.g., acute osteomyelitis, nosocomial lower respiratory tract infections, complicated urinary tract infections, bacterial meningitis, and bacteremia [64]
Daptomycin	Cyclic lipopeptide core of 13 amino acids, results in membrane depolarization and subsequent loss of intracellular components [50]/altered membrane composition [6]	Intrinsic resistance to daptomycin in Gram-positive bacteria, daptomycin resistance gene *mprF* acquired resistance among *Enterococcus* spp. [50]	Daptomycin may also penetrate immune cells including neutrophils and macrophages—immunomodulatory [50], first-line agent to treat severe VRE infections, antibiofilm activity [50]

LPS—lipiopolysaccharide, BSI—bloodstream infection, UTI—urinary tract infection, VRE—vancyomcin resistant *Enterococci*, ESBL—extended-spectrum beta-lactamase, CAP—community-acquired pneumonia.

### 2.2. AMP Activity against Gram-Negative Pathogens Associated with Infectious Disease

Gram-negative pathogens are abundant on the World Health Organization (WHO) priority pathogen list due to their virulence and high levels of AMR. Critical Gram-negative pathogens include *Acinetobacter*, *Pseudomonas*, *Klebsiella*, *E. coli*, and *Proteus* species among other members of the Enterobacterales. ESBLs producing Gram-negative pathogens possess intrinsic resistance to most β-lactam antibiotics, including penicillin and cephalosporins. Gram-negative pathogens are often associated with BSIs and pneumonia, where pneumonia caused by MDR species is associated with high morbidity and high mortality rates, particularly in ICU patients [30]. The presence of efflux pumps and the cell membrane among other AMR traits imparts excellent antibiotic resistance on Gram-negative species. The outer membrane of Gram-negative bacteria contains LPS, which provides a robust barrier inhibiting antibiotics from entering the cell to affect cell components such as nucleic acids [3]. This membrane provides a permeability barrier and impedes the activity of antibiotics, compounding therapy in conjunction with MDR [65]. The LPS, once released from the cell membrane during cell reproduction or cell death, is associated with sepsis, mortality, and morbidity in patients by stimulating immune responses and the release of inflammatory cytokines, e.g., TNF-α [53]. AMPs, which bind to the LPS moiety of the cell membrane, result in a loss of membrane structural integrity resulting in cell death. The last-resort antibiotic and AMP colistin is applied for Gram-negative pathogens and has demonstrated resistance to frontline antibiotics, including β-lactams, aminoglycosides, and fluoroquinolones.

AMPs have demonstrated efficacy against many clinically relevant Gram-negative pathogens. Nisin and carnocyclin A demonstrated activity against Gram-negative pathogens when combined with chelating agents, e.g., ethylenediaminetetraacetic acid (EDTA) allowing for penetration of the cell membrane [11]. Mutacin B-Ny266 a lantibiotic has activity against *Neisseria* and *Helicobacter* species with klebicins and has demonstrated activity against MDR and carbapenem-resistant *Klebsiella* species [46]. This AMP also proved effective against *S. aureus* in infected mice [46]. Studies report computationally designed/synthetic AMPs termed PHNX-1, 7, and 8, which have demonstrated activity against Gram-negative bacteria at 100 μg/mL [65]. The synthetic AMP murepavadin, which alters the bacterial cell membrane produced by the Swiss company Polyphor AG, reached phase III clinical trials against *Pseudomonas aeruginosa* infection by IV administration but was ceased in 2019 due to renal toxicity [22]. Murepavadin has entered phase I trials but as an inhalation formulation against *P. aeruginosa* in cystic fibrosis patients [22]. Bacteriocin pyocin SD2, S2, and S5 produced by *E. coli* demonstrated potent activity against *P. aeruginosa* in vivo using a mouse model [46]. Proline-rich insect AMPs appear more toxic to Gram-negative pathogens with pyrrhocoricin and apidaecins (proline-rich AMPs) demonstrating efficacy against *E. coli* [24]. Pyrrhocoricin and apidaecins are insect AMPs produced by Pyrrhocoris apterus and Apis melifera, respectively, and have potent activity against Gram-negative bacterial strains [24]. Importantly, the AMPs in development and under Food and Drug Administration (FDA) approval have not demonstrated potency against Gram-negative species comparative to Gram-positive species [51]. The AMPs pexiganan and LL-37 result in haemolytic damage at their MIC range (1.7 to 16 µM), thus limiting their application to topical treatments [51]. Studies show that LL-37 has an anti-LPS toxin effect as determined via in vivo rat models [52]. Studies have described the ability of AMPs to neutralize or inhibit LPS and to reduce LPS inflammation with the AMP HV2 inhibiting LPS cytokine induction [53]. The amphibian AMP citropin 1.1 significantly reduced plasma endotoxins and inflammatory cytokines in *E. coli*-induced sepsis [55]. The broad-spectrum citropin 1.1 is produced in the skin of an Australian tree frog. Tachyplesin III produced by the horseshoe crab is a potent disulphide-linked peptide and demonstrated activity in vitro when combined with beta-lactams and colistin against MDR strains of *P. aeruginosa* [55]. The synthetic AMC-109 demonstrated activity against *P. aeruginosa* with an MIC of 8–16 µg/mL [43]. The AMPs LGL13K and DGL13K modified from a salivary AMP have demonstrated activity against AMR Gram-negative pathogens, including ESBL *K*. *pneumoniae* with an MIC range of 16–32 μg/mL, MDR and XDR *P*. *aeruginosa*, and XDR *A*. *baumannii* carrying metallo-beta-lactamases with an MIC of 8–32 μg/mL [66]. The synthetic AMP IB-367 in combination with colistin and imipenem inhibited *P. aeruginosa* and *E. coli* in mouse dermal wounds [55]. The AMP pepW is effective against the capsule of *K. pneumoniae* with an MIC between 2 and 4 µg/mL and against *E. coli* with an MIC of 1–2 µg/mL [25].

### 2.3. AMP Resistance

Resistance to AMPs, while not impossible, is not as common as resistance to conventional antibiotics. AMPs are typically faster acting than antibiotics, thus limiting the time for resistance to emerge [67]. AMPs are degraded by proteases and peptidases post activity; they have a non-specific mode of action (multi hit mode of action) and genetic mutations, which impart resistance and tend to come with a fitness cost [52]. Alteration of the bacterial membrane or surface remodeling to reduce the overall negative charge is a mechanism of resistance to AMPs observed in many *Bacillus* species, *L. monocytogenes*, *C. difficile*, and *Lactobacillus* species [14]. Bacterial strains having resistance to polymyxins, cathelicidins, and defensins display altered membrane lipid composition with lower levels of certain membrane proteins and ions [68]. Alteration of cell wall components (target site alteration), lipid composition, efflux pumps, presence of capsules, and secretion of proteases, e.g., aureolysin, also imparts resistance mechanisms in Gram-positive species [14]. The Gram-negative *Pseudomonas* also displays many of such resistance mechanisms [43]. Intrinsic and acquired resistance to colistin is associated with modification of the LPS membrane components (target site alteration), efflux pumps, and production of capsules in Gram-negative species [37], and it is becoming increasingly common in clinical settings.

**Figure 2:**
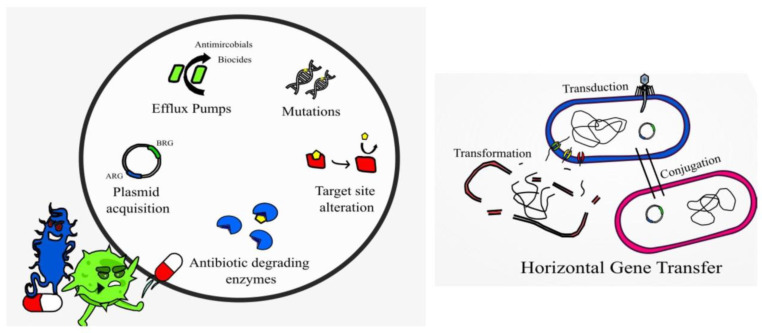
Displaying AMP-resistance mechanisms present in certain bacterial species.

Interestingly, studies have described the use of AMPs to block or inhibit the action of efflux pumps, such as the small multidrug resistance (SMR) efflux pump [68]. Alarmingly, resistance to colistin in *A. baumannii* is due to a complete loss of LPS from the cell membrane [69]. Bacteria may also produce and secrete non-proteolytic AMP-sequestering proteins, and these bind the AMP preventing their action, e.g., the enzyme staphylokinase produced by *S. aureus* [68]. Research studies demonstrate that sublethal doses of AMPs melittin and pexiganan prime bacterial cells to increase both tolerance and persistence as demonstrated with *E. coli* [70]. Melittin has demonstrated anticancer activity, which is non-specific resulting in cytotoxicity and hemolytic activity [44]. Resistance to bacteriocins may occur when bacteria mimic the defense mechanisms of the producer strain, adapt the cell membrane or via enzymatic activity in a similar manner to antibiotic resistance [11] (Figure 2). For example, bacteria may have resistance to nisin by producing dehydropeptide reductase, or nisinase to deactivate the bacteriocin [9]. Augmenting antibiotic therapy with AMPs may hinder AMP resistance and provide effective antibacterial action [43]. Genetically engineering peptides to contain a higher content of key antimicrobial amino acids may also prevent AMP resistance and provide more potent broad-spectrum action [67].

## 3. Issues Preventing the Application of AMPs as Broad-Spectrum Antimicrobials

Currently, AMPs are not broadly implemented in the treatment of infectious disease with the exception of colistin and gramicidin. Their biocompatibility and cytotoxicity in vivo greatly hinder their application parenterally, with nephrotoxicity and hepatoxicity being evident in some cases [44,54]. Indeed, the AMPs currently in use are considered last-resort antibiotic agents [6,50]. Issues with large-scale production and drug formulation to meet the needs of large populations are also present, where the mass production of antibiotic agents is a well optimized and a more efficient system is required to produce the quantities needed at global level. The sporicidal [11], antibiofilm [25,42,50] and potent activity against AMR species means that AMPs warrant extensive research to overcome such issues.

### 3.1. Future Direction of AMP Production and Formulation to Overcome Current Issues

The AMP database gives a list of the current natural and synthetic peptides that have been identified. Defensins and bacteriocins are the groups that show highest antimicrobial potential for application as human therapeutics [46]. The large-scale production and clinical application of AMPs are areas of much research with in vitro studies, showing the promising potential of these potent peptides. The production of sufficient quantities of suitable purity remains a challenge to clinical application, with isolation from natural sources providing low AMP yield [71]. The use of solid-phase peptide synthesis allows for AMP synthesis of peptides of small to medium size (50 amino acids) [68] but scale up is not feasible and its suitability for producing long chain peptides is limited and results in sequence errors [71]. Natural AMPs are prone to protease degradation due to the L-amino acid content and results in poor bioavailability when administered therapeutically. The genetic engineering of AMPs using recombinant DNA technology and expressions systems grown in bioreactors including bacterial, yeast, plant, or animal cells may allow for large-scale production. Bioreactors allow for critical parameter control such as temperature, pH, and dissolved oxygen (DO) to obtain optimal cell density and protein yield [71]. Currently, synthesis methods have resulted in low yield and downstream processing issues with poor quality AMP production limiting the number of AMP reaching clinical trials and market [72]. The bacterial species *E. coli* and *B. subtilis* and yeast *Pichia pastoris* and *Saccharomyces cerevisiae* have been utilized for the production of biologics and AMPs due to their fast growth rate, cheap media requirements, and high yields [73]. These yeast expression systems are used to produce AMPs, including cathelicidin, enterocin, pediocin, plantaricin, and α-sarcin, while *E. coli* and *B. subtilis* have been used to produce defensin, hepcidin, histonin, and lactoferrin among others [74]. Yeast expression systems are particularly beneficial as they are robust, readily agreeable to genetic engineering/modification (GM), cost-effective, and able to carry out post-translational modifications (PTMs), with no endotoxin production that may contaminate the production process, as seen with bacterial systems [75]. Endotoxin contamination, which can occur with bacterial expression systems, may result in fatal septic shock in treated patients [76].

Post-translational modification, which is the alteration of the peptide via the addition of a chemical group such as a carbohydrate (glycosylation) or peptide (ubiquitylation), is key to the functioning of biologics in vivo [75]. C-terminal amidation appears important for antimicrobial activity because it raises the net charge of a peptide through neutralization of the C-terminal carboxylate and the helicity of the peptide [68]. Studies show that amidated AMPs repeatedly have the lowest MIC values [77]. Lantibiotics, which are ribosomal-synthesized peptides, are post-translationally modified via glycosylation [78], while class II bacteriocins do not have large PTM needs [46]. The toxicity of AMPs to host microbial expression systems is an issue with bacterial and yeast-based production systems [76]. Plant chloroplasts such as AMP expression systems in bioreactors show potential due to their high yield because each plant cell has numerous chloroplasts. The expression of AMPs in plants has many benefits, including cheaper cost, high yield, ease of scale up, reduced purification and processing steps, low contamination issues, increased biocompatibility of the product, and ability to conduct PTMs [74]. Smaller peptides (<65 amino acids) produced in plant chloroplasts, however, are prone to protease degradation [72]. Research shows reduced protease activity by constructing protein fusions that produced larger AMPs that were not recognized by plant protease enzymes by linking or fusion smaller AMPs together [79]. Producing fusion AMPs aids in production, purification, and reduces proteolysis by expression systems because it increases the overall size of the AMP [71]. Cleavage of the fusion tag can then be achieved via enzymatic or chemical means prior to formulation [71]. The human-derived cathelicidin antimicrobial peptide (hCAP18/LL-37) has been expressed in the Chinese cabbage plant and LL-37 in Hordeum vulgare L. (Barley) [72]. The AMP Protegrin-1 has been expressed in a tobacco plant showing efficacy against *K. pneumoniae*, *S. aureus*, *E. coli*, and *C. albicans* [74]. Long AMP sequences tend to have high production costs, issues with enzymatic degradation, and induce immunogenic reactions in vivo, and trimming unnecessary amino acid sequences or regions may shorten the AMP and reduce this production limitation [67]. The use of edible plants as expression systems may double as an oral mode of delivery and thus eliminate the need for downstream processing, gastrointestinal degradation, and risk of septic shock in the patient [76]. Research studies successfully produced a cecropin-like insect AMP (MIC of 0.8 µM for *E. coli*) using a cell line derived from an insect (armyworm moth) in a continuous process, with the isolated product having efficacy against *E. coli* [77]. Recombinant AMP production has a reduced cost and lower environmental impact [74] but is more complex, often requiring cleavage of fusion tags at purification, and is more labor intensive than chemical synthesis [68]. Synthetic peptides can also be produced by ring opening polymerization (ROP) of N-carboxyanhydrides derived from a-amino acids (NCAs); an excellent review of this process is provided by Rasines Mazo et al., (2020) [80].

### 3.2. Pharmacokinetics and Pharmacodynamic Considerations

The use of AMPs therapeutically is susceptible to formulation limitations due to their pharmacodynamic and pharmacokinetic profiles in vivo, which impacts their route of administration. Orally delivered AMPs are prone to protease degradation in the GIT, chemical instability, and adsorption issues limiting their bioavailability, while some are pH sensitive (Table 3) [67]. Parenterally administered formulations avoid GIT degradation but may be exposed to proteases present in the bloodstream and binding to circulating serum albumin [46].

Furthermore, AMPs have reduced antibacterial activity in vivo due to physiological salt impacting on the electrostatic interactions with cell membranes [9]. This instability of AMPs greatly affects their pharmaceutical development, formulation, and clinical use. To allow for oral delivery and improved bioavailability of AMPs, the use of drug delivery systems can be employed. Nisin is readily degraded in the GIT and so has been encapsulated in pectin-based compression coated tablet, giving a controlled-release delivery system [9]. Encapsulation within delivery systems composed of synthetic polymers, polysaccharides, proteins, liposomes, or inorganic materials improves the immunogenicity, biocompatibility, and stability of peptide therapeutics [81]. Using biocompatible polymers as delivery systems for AMPs can improve in vivo stability, half-life, and reduce cytotoxicity [44]. Encapsulation in nanoparticle delivery systems may improve the targeting of intracellular pathogens such as clinically relevant *M. tuberculosis*, *S. enterica,* and *L. monocytogenes* [68]. The failure of the macrolide murepavadin to pass phase III clinical trials as an IV administered antibiotic due to renal toxicity prompted its phase I investigation for inhaled treatment of *P. aeruginosa* lung infection. Murepavadin demonstrated a good pharmacokinetic and safety profile in healthy volunteers at up to 300 mg, with further testing to follow at phase II [82].

The formulation of AMPs as prodrugs may improve the bioavailability of orally delivered peptides. Prodrugs are inactive formulations that are activated in vivo biochemically/chemically to allow for targeted drug delivery. An AMP prodrug may be constructed via linking the peptides to a promoiety such as an amino acid that is cleaved via protease activity in the GIT or via the pathogen itself [68]. Biocompatibility issues relating to destruction of the host cell membrane such as erythrocyte cells leading to hemolysis [51] and cell death by AMPs have been observed in vivo [53], which prohibits systemic application clinically. Genetically engineering the AMPs to alter peptide amino acid sequences to increase antibacterial activity and selectivity to protect host cells may help overcome such issues [53]. Similarly, genetically modifying AMPs by altering amino acid sequences to be less susceptible to proteolytic degradation in vivo may improve bioavailability [46]. At present, there is a sparsity of human in vivo studies detailing the biocompatibility profile of AMPs with studies currently limited to cytotoxicity and hemocompatibility. In accordance with the FDA and International Standards Organization (ISO), testing guidelines testing the sensitization, pyrogenicity, genotoxicity, reproductive toxicity, and more is required to achieve FDA approval [44].

## 4. Conclusions

The emergence and proliferation of antibiotic resistant bacterial species are having a drastic impact on disease treatment, morbidity, and mortality. The search for alterative or combination therapeutics is ongoing with antimicrobial peptides showing potent antibacterial action in vitro. Compared to traditional antibiotics, AMPs have many advantages including their multi-hit mode of action, immune-stimulating activity, and rapid onset of bactericidal activity leading to low levels of resistance. Resistance mechanisms do exist, however, including efflux pumps, enzyme excretion, membrane alteration, and studies are warranted to investigate the full potential of AMP resistance to emerge post therapeutic application. Furthermore, AMPs have demonstrated antibiofilm activity, allowing for their implementation as coatings on medical devices preventing infectious disease. Traditionally, the production of AMPs was achieved via solid-phase peptide synthesis with limited yield of small to medium peptides. The use of RDNA technology and prokaryotic and eukaryotic expression systems has limitations, including low yield, protease degradation in situ, expression cell toxicity, and purification issues. Additionally, the clinical application of AMPs remains hindered due to their inherent toxicity to host cells, including erythrocytes, pharmacokinetic and pharmacodynamic issues, stability issues in vivo, and large-scale production costs. Formulating AMP therapeutics encapsulated in biocompatible polymers or as prodrugs may overcome such administration issues. Augmenting antibiotics with AMPs may allow for a therapeutic effect at lower antibiotic concentrations and sub-MIC AMP concentrations effective against MDR pathogens. There is, however, a scarcity of clinical trials assessing the efficacy of such combination formulations.

## Figures and Tables

**Table 3:** Advantages, disadvantages, and limitations of antimicrobial peptides.

Advantages	Disadvantages	Limitations
Some AMPs show synergistic interactions with conventional antibiotics [9]	Cytotoxic/biocompatibility issues	Unfavorable pharmacokinetic profile—may be improved by formulating as a prodrug
Broad-spectrum of antimicrobial activity against yeast, fungi, viruses, and bacteria	Bacterial resistance may emerge to certain AMPs	No clear in vivo efficacy over conventional treatments [9]
Easier to synthesize—short amino acid sequences	Limited stability	Some require PTMs limiting expression systems [67]
Rapid onset of action	Short half-life	Downstream purification issues post production
Potent	Protein and enzymatic degradation	Formulation for oral delivery raises issues
Not effected by AMR phenotypes [25]	Reduced in vivo antimicrobial action	Not usually tolerant of low-pH environments
Some effective against biofilms	Over stimulation of immune system may be an issue	May lose activity in the presence of physiological salts or serum [24]
Potential for use as vaccine adjuvants [25]	Expensive to produce [67]	Binding to serum proteins such as albumin [67]
Some AMPs are stable and active in a wide pH range [6]	Toxicity to microbial expression systems during production [76]	Limited in vivo biocompatibility information currently available [44]
May not induce dysbiosis in the patient [38]	May induce pro-inflammatory cytokines [25]	
Immunomodulatory effects		
AMPs can self-assemble in to various structures which may aid potency [42]		

AMP—antimicrobial peptide, PTM—post-translational modification, AMR—antimicrobial peptide.

## Data Availability

Not applicable.
